# Endothelial Progenitor Cells Produced From Human Pluripotent Stem Cells by a Synergistic Combination of Cytokines, Small Compounds, and Serum-Free Medium

**DOI:** 10.3389/fcell.2020.00309

**Published:** 2020-05-15

**Authors:** Simon Farkas, Pavel Simara, Daniela Rehakova, Lenka Veverkova, Irena Koutna

**Affiliations:** ^1^Department of Histology and Embryology, Theoretical Departments, Faculty of Medicine, Masaryk University, Brno, Czechia; ^2^International Clinical Research Center, St. Anne’s University Hospital Brno, Brno, Czechia; ^3^I. Surgery Department, St. Anne’s University Hospital Brno, Brno, Czechia

**Keywords:** hiPSC, hESC, hPSC, mesoderm, endothelial progenitors, differentiation, protocol

## Abstract

Human pluripotent stem cells (hPSCs) are a promising source of autologous endothelial progenitor cells (EPCs) that can be used for the treatment of vascular diseases. However, this kind of treatment requires a large amount of EPCs. Therefore, a highly efficient, robust, and easily reproducible differentiation protocol is necessary. We present a novel serum-free differentiation protocol that exploits the synergy of multiple powerful differentiation effectors. Our protocol follows the proper physiological pathway by differentiating EPCs from hPSCs in three phases that mimic *in vivo* embryonic vascular development. Specifically, hPSCs are differentiated into (i) primitive streak, which is subsequently turned into (ii) mesoderm, which finally differentiates into (iii) EPCs. This differentiation process yields up to 15 differentiated cells per seeded hPSC in 5 days. Endothelial progenitor cells constitute up to 97% of these derived cells. The experiments were performed on the human embryonic stem cell line H9 and six human induced pluripotent stem cell lines generated in our laboratory. Therefore, robustness was verified using many hPSC lines. Two previously established protocols were also adapted and compared to our synergistic three-phase protocol. Increased efficiency and decreased variability were observed for our differentiation protocol in comparison to the other tested protocols. Furthermore, EPCs derived from hPSCs by our protocol expressed the high-proliferative-potential EPC marker CD157 on their surface in addition to the standard EPC surface markers CD31, CD144, CD34, KDR, and CXCR4. Our protocol enables efficient fully defined production of autologous endothelial progenitors for research and clinical applications.

## Introduction

According to the World Health Organization, ischemic heart disease and stroke have been two major causes of death worldwide for the last 15 years. It is therefore very desirable to find an efficient treatment for such devastating diseases. Both diseases are often the result of worn-out and/or damaged endothelial cells (ECs). Replacement of dysfunctional ECs with healthy young ECs seems to be a logical solution that will be applicable in the foreseeable future. However, it is first necessary to determine which subtype of ECs is best suited for this job, and second, we have to produce this subtype of ECs in high-enough numbers to treat the aforementioned diseases. Last but not least, this cell production needs to be both robust and standardized, if it is to be ever widely used in clinical practice.

Endothelial cells form the linings of blood and lymphatic vessel lumens. They have regulatory roles in physiological processes, such as maintaining vascular tone and homeostasis; they participate in angiogenesis and vasculogenesis, and they mediate interactions of the vessel wall with blood elements (Carmeliet, 2000). The ECs most commonly found in the human vasculature are mature ECs such as human saphenous vein ECs (HSVECs) and human umbilical vein ECs (HUVECs), which can be harvested from umbilical cords, as their name suggests. Mature ECs highly express the surface pan-endothelial markers CD31 and CD144. CD34 and kinase insert domain receptor (KDR), which are mostly associated with the progenitor status of ECs, can also be expressed on the surface of mature ECs, although their expression is dim to none. Endothelial cells grow in cobblestone formation and have proliferative capacity. Nonetheless, mature ECs lack angiogenic and vasculogenic properties, which significantly reduces their potential use in regenerative medicine.

Endothelial progenitor cell (EPC) is a general term for a group of cells defined by high surface expression of the markers CD31, CD144, CD34, and KDR in the entirety of their populations. This group contains primitive ECs with improved angiogenic and vasculogenic properties (Cheng et al., 2013; Patel et al., 2013, 2017; Lee et al., 2015; Shafiee et al., 2018). These primitive ECs, also known as late EPCs or endothelial colony-forming cells (ECFCs), are derived from mesoderm, and they have the ability to proliferate and to differentiate into mature ECs. They grow in cobblestone formation such as mature ECs, and they cannot be distinguished from these cells by light microscopy. A significant fraction of ECFCs expresses C-X-C chemokine receptor type 4 (CXCR4/CD184) on their surface in addition to other EPC surface markers (Joo et al., 2015; Kang et al., 2015). While CXCR4 is not expressed in mesoderm, it is later expressed in some of its progeny, including ECFC. Presence of CXCR4 on the cell surface improves homing capabilities, which is desirable property in progenitor cells. Endothelial colony-forming cells contain a subpopulation of very proliferative, angiogenic, and vasculogenic cells, which are referred to as high-proliferative-potential (HPP) ECFCs. This subpopulation was recently discovered to express bone marrow stromal cell antigen 1 (CD157/BST-1) on their surface, unlike any other endothelial population reported so far (Wakabayashi et al., 2018). Because of their properties, ECFCs and HPP-ECFCs are ideal candidates for use in regenerative medicine.

More than 150 clinical trials are currently being conducted on ECFCs, mainly to treat myocardial infarction and peripheral vascular disease (Chong et al., 2016). Such treatments require vast amounts of ECFCs to be successful. However, ECFCs from blood vessels or peripheral blood can only be obtained in limited numbers, which makes it impossible to expand them to sufficient numbers without compromising their proliferative potential. To overcome these hurdles, human pluripotent stem cells (hPSCs) can be differentiated into endothelium. Attempts at efficient *in vitro* endothelial differentiation of hPSCs have been conducted for at least 10 years (Choi et al., 2009; Park et al., 2010; Vodyanik et al., 2010; Joo et al., 2011; Li et al., 2011; Tatsumi et al., 2011; Adams et al., 2013; Prasain et al., 2014; Sahara et al., 2014; Zhang et al., 2014; Bao et al., 2015; Patsch et al., 2015; Sriram et al., 2015; Kitajima et al., 2016; Ye et al., 2016; Harding et al., 2017; Olmer et al., 2018; Suknuntha et al., 2018; Zhao et al., 2018). This strategy has the potential to ensure a consistent and unlimited source of ECFCs for *in vitro* studies and regenerative medicine. There are two major approaches to endothelial differentiation of hPSCs. First, embryoid body-based differentiation may be used, but it is a time-consuming and relatively inefficient method of endothelial derivation (Li et al., 2011; Adams et al., 2013). Second, monolayer differentiation is a more feasible approach, with higher efficiency in a shorter time. There are multiple monolayer differentiation protocols that vary in both medium and cytokine supplement (Park et al., 2010; Joo et al., 2011; Tatsumi et al., 2011; Orlova et al., 2014; Prasain et al., 2014; Sahara et al., 2014; Bao et al., 2015; Patsch et al., 2015; Sriram et al., 2015; Kitajima et al., 2016; Harding et al., 2017; Zhao et al., 2018). Most monolayer protocols use single-cell seeding, small-clump seeding using ethylenediaminetetraacetic acid (EDTA), or larger-clump seeding using a needle. In general, clump-based differentiation protocols have higher differentiation efficiency than single-cell–based protocols. Unfortunately, clump-based protocols have very low reproducibility as they are highly dependent on the individual skill of the operator, and they make it harder to quantify the number of cells used. In contrast, single-cell protocols enable usage of more precise amounts of cells, which makes them potentially more suitable for standardized procedures, should their lower efficiency be resolved. A highly efficient, robust and standardized protocol is necessary in order to differentiate large-enough amounts of ECFCs from hPSCs, to satisfy the needs of regenerative medicine. We hypothesized that the best way to produce these ECFCs is by a differentiation protocol that replicates the three most important naturally occurring steps of embryonic endothelial differentiation. These steps are as follows: (i) rise of the primitive streak from the epiblast, (ii) differentiation of the primitive streak into mesoderm, and (iii) differentiation of the mesoderm into blood islands (a population of endothelial progenitors). To efficiently replicate these steps *in vitro*, we used known effectors of endothelial differentiation (Sahara et al., 2014; Patsch et al., 2015; Sriram et al., 2015; Kempf et al., 2016) in a novel synergistic strategy.

**FIGURE 1:**
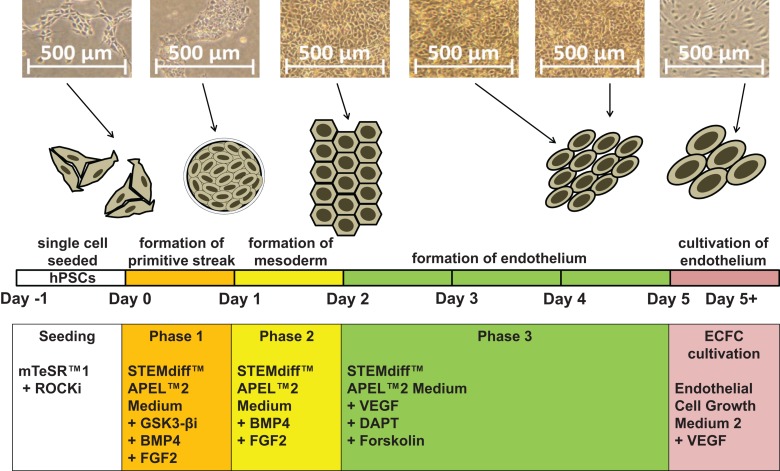
Schematic description of our synergistic three-phase differentiation protocol. Representative photographs of the hiPSC line CBIA-50 differentiated by our three-phase protocol and schematic description of this differentiation protocol. Human pluripotent stem cells (hPSCs) are seeded in single-cell format and then differentiated into endothelium in three phases. Lastly, the derived ECFCs are further cultivated.

Our new protocol has three phases, and the unique medium composition in each phase drives the transition of hPSCs first to the primitive streak and then to KDR^+^ mesoderm and finally to primitive endothelium (Figure 1). With our synergistic three-phase protocol, we were able to differentiate all seven hPSC lines used in this study into endothelium in only 5 days, with very high efficiency under standardized and fully described conditions. The derived endothelium fitted the description of HPP-ECFCs. To our knowledge, this is the most robust endothelial differentiation experiment conducted up to this point, given that the hPSCs used included a human embryonic stem cell (hESC) line and six human induced pluripotent stem cell (hiPSC) lines created by three different methods from multiple donor cell types. Therefore, our synergistic three-phase protocol is fully replicable and highly efficient and produces cells that fit the most recent profile of endothelial progenitors (HPP-ECFCs).

## Materials And Equipment

### Cytokines and Small Compounds

1. Y-27632 2HCl (ROCK1 inhibitor; Selleckchem, Houston, TX, United States, S1049);
2. CP21R7 [glycogen synthase kinase 3 beta (GSK3-β) inhibitor; Selleckchem, S7954];
3. BMP4 (Peprotech, Rocky Hill, NJ, United States, 120-05ET);
4. FGF2 (Peprotech, 100-18B);
5. VEGF_165_ (Peprotech, 100-20);
6. DAPT–γ-secretase inhibitor (Sigma–Aldrich, St. Louis, MO, United States, D5942-5MG);
7. Forskolin (Sigma–Aldrich, F3917-10MG).

### Other Reagents and Media

1. mTeSR1^TM^ (STEMCELL Technologies, Vancouver, BC, Canada, 85850);
2. Corning Matrigel matrix (Corning, Corning, NY, United States, 354277);
3. TrypLE^TM^ Express (1×; Thermo Fisher Scientific, Waltham, MA, United States, 12604021);
4. STEMdiff^TM^ APEL^TM^2 (STEMCELL Technologies, 5270);
5. Fibronectin (Sigma–Aldrich, F0895-5MG);
6. Endothelial cell growth medium 2 (PromoCell, Heidelberg, Germany, C-22011);
7. Phosphate-buffered saline (PBS), pH 7.4 without phenol red, calcium or magnesium (Thermo Fisher Scientific, 10010056);
8. UltraPure^TM^ 0.5M EDTA, pH 8.0 (Thermo Fisher Scientific, 15575020);
9. Bovine serum albumin (BSA; Sigma, A4503);
10. ZellShield^®^ (Minerva Biolabs, Berlin, Germany);
11. DMEM/F-12, no glutamine (Thermo Fisher Scientific, 21331020).

### Florescence-Activated Cell Sorting Antibodies and Low-Density Lipoprotein

Combine two different types of antibodies (APC-conjugated + PE-conjugated antibodies) per sample if possible.

1. Anti-CD31 antibody (allophycocyanin [APC] conjugated; AC128; Miltenyi Biotec, Bergisch Gladbach, Germany, 130-092-652);
2. Anti-CD34 antibody (phycoerythrin [PE] conjugated; AC136; Miltenyi Biotec, 130-081-002);
3. Anti-CD144 antibody (PE conjugated; REA199; Miltenyi Biotec, 130-100-708); alternatively, the APC- conjugated variant may be used for convenience;
4. Anti-KDR antibody (PE conjugated; ES8-20E6; Miltenyi Biotec, 130-093-598);
5. Anti-CXCR4 antibody (APC conjugated; REA649; Miltenyi Biotec, 130-098-357);
6. Anti-CD157 antibody (APC conjugated; REA465; Miltenyi Biotec, 130-106-982);
7. Dil-labeled and acetylated (Dil-Ac)-LDL (Alpha Diagnostics, San Antonio, TX, United States, LDLA16-N-1).

### Equipment and Software

1. BD FACS Canto II flow cytometer (Becton–Dickinson, Heidelberg, Germany);
2. BD FACSDiva analysis software (Becton–Dickinson);
3. Flowing software (Cell Imaging Core, Turku Centre for Biotechnology, Turku, Finland);
4. CD31 MicroBead Kit (Miltenyi Biotec, 130-091-935);
5. CD144 MicroBead Kit (Miltenyi Biotec, 130-097-857);
6. MiniMACS^TM^ Separator (Miltenyi Biotec, 130-042-102);
7. MS Column (Miltenyi Biotec, 130-042-201).

### Media Recipes

1. hPSC cultivation medium: mTeSR-1 medium;
2. Predifferentiation medium: mTeSR-1 medium and 10 ng/mL Y-27632 2HCl (ROCK1 inhibitor);
3. Phase 1 medium: STEMdiff APEL2, 3 μM CP21R7 (GSK3 -β inhibitor), 25 ng/mL BMP4, and 50 ng/mL FGF2;
4. Phase 2 medium: STEMdiff APEL2, 25 ng/mL BMP4 and 50 ng/mL FGF2;
5. Phase 3 medium: STEMdiff APEL2, 200 ng/mL VEGF_165_, 10 μM DAPT, and 2 μM forskolin;
6. ECFC cultivation medium: endothelial cell growth medium 2 and 50 ng/mL VEGF_165_.

Notes: To prevent contamination, all media used in the experiment contained Zell Shield (Minerva Biolabs) in a 1:100 ratio (according to manufacturer instructions). All supplements were mixed with their respective media according to manufacturer instructions.

### Dish Coating

Dishes for hPSC cultivation and differentiation: ice-cold Matrigel was mixed with ice-cold DMEM2/F-12, no glutamine according to manufacturer instructions (Corning, 354277; the exact ratio was LOT dependent). Dishes were coated at 1 mL/10 cm^2^ and left at room temperature for at least 1 h prior to use.

Dishes for somatic ECs and derived ECFCs: Refrigerated (8°C) fibronectin was mixed with refrigerated (8°C) PBS at a 1:40 ratio. Dishes were coated at 1 mL/10 cm^2^ (2.5 μg/cm^2^) and left at room temperature for at least 1 h prior to use.

### Cultures of hPSCs Used to Test Efficiency and Robustness of the Protocol

The six hiPSC lines used were generated and characterized in our laboratory (Simara et al., 2014; Tesarova et al., 2016), and the hESC H9 (WA09) line was bought from WiCell Research Institute (Madison, WI, United States). The hPSC cultures were maintained long term in hPSC cultivation medium on hPSC cultivation dishes according to instructions from their respective manufacturers (STEMCELL Technologies 85850 and Corning 354277).

#### hPSC ID, Stem Cell Type, Source Cell Type, and Reprogramming Methods

1. H9 (WA09), hESC, human embryo, no reprogramming method;
2. CBIA-3, hiPSC, CD34^+^ blood progenitor, Sendai virus;
3. CBIA-7, hiPSC, human adult dermal fibroblasts, episomal vector;
4. CBIA-19, hiPSC, HUVECs, episomal vector;
5. CBIA-37, hiPSC, HSVECs, episomal vector;
6. CBIA-50, hiPSC, HUVECs, StemRNA-NM^TM^ Reprogramming Kit;
7. CBIA-58, hiPSC, HSVECs, StemRNA-NM^TM^ Reprogramming Kit.

#### Somatic Endothelial Cells Used in the Experiment

1. HUVEC1—cell line pooled from multiple donors and bought from Thermo Fisher Scientific;
2. HUVEC2—cell line isolated from single donor in our laboratory;
3. C2—a HSVEC line from single donor isolated in our laboratory.

## Procedures

### Predifferentiation (Up to Day -1)

Maintain the hPSC cultures in hPSC cultivation medium on hPSC cultivation dishes for at least three passages prior to differentiation. Upon reaching 70 to 80% confluence, add 10 ng/mL Y-27632 2HCl to the medium for at least 1 h. After this exposure to Y-27632 2HCl, dissociate hPSCs into single cells using TrypLE according to the manufacturer instructions. Seed the dissociated cells at a density of 40,000 cells/cm^2^ and culture them in predifferentiation medium for 1 day (day -1).

### Phase 1 (Day 0)

Remove predifferentiation medium and wash cells with PBS. Remove PBS and add phase 1 medium, using 1 mL/10 cm^2^ of culture dish, and culture the cells in these conditions for 1 day.

### Phase 2 (Day 1)

Exchange phase 1 medium for phase 2 medium, using 2 mL/10 cm^2^ of culture dish, and culture the cells in these conditions for 1 day.

### Optional Mesoderm Verification (Day 2)

On day 2, collect adherent differentiating cells by TrypLE and analyze them by florescence-activated cell sorting (FACS) for the mesoderm-specific marker KDR and the endoderm-specific marker CXCR4. KDR should be highly expressed, whereas CXCR4 should have no or only dim expression in a small portion of the cell population, as exemplified in Figure 3A.

### Phase 3 (Days 2–4)

Exchange phase 2 medium for phase 3 medium on day 2, using 2 mL/10 cm^2^ of culture. On days 3 and 4, replace old phase 3 medium with fresh phase 3 medium.

### End of Differentiation (Day 5)

**FIGURE 2:**
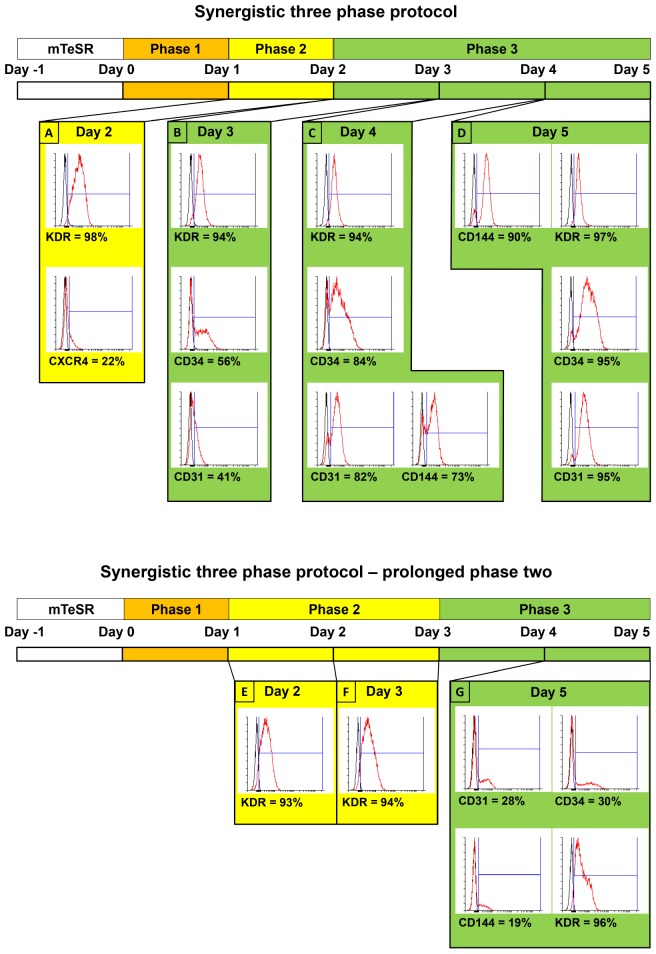
Comparison of surface marker expressions between two variants of synergistic three-phase protocol, including simplified schemes of both variants. Both variants were tested using the CBIA-50 hiPSC line. The first variant is the one that is fully described in this article. Stage 2 lasts for only 1 day, whereas stage 3 lasts for 3 days. **(A)** KDR is expressed on the surface of 98% of cells, whereas CXCR4 is dimly expressed on 22% of cells by day 2 of differentiation. **(B–D)** KDR remains highly expressed during phase 3 of differentiation (94–97%), and other endothelial markers start to emerge by day 3 and gradually increase until they reach very high expression (90%–95%) by day 5 of differentiation. **(B)** Second variant of our protocol, in which both phases 2 and 3 last for 2 days. **(E,F)** Similar to the previous variant, KDR is expressed on 94% of cells on days 2 and 3. **(G)** By the end of phase 3 on day 5 of differentiation, the KDR expression is slightly reduced to 87%. More importantly, other endothelial markers, CD31, CD34, and CD144, have expression of only 28, 30, and 19%, respectively.

On day 5, collect adherent differentiated cells by TrypLE and analyze a fraction of them by FACS for the endothelial surface markers CD31, CD144, CD34, and KDR (Figure 2D). Cell cultures with ≥85% expression of either CD31 or CD144 can be seeded without any separation. If the expression of both CD31 and CD144 in derived cells is <85%, separation by magnetic-activated cell sorting (MACS) for the marker CD31 or CD144 is necessary prior to seeding. Next, seed the differentiated cells at a density of 10,000 cells/cm^2^ on dishes for derived ECFCs and cultivate them in 2 mL ECFC cultivation medium/cm^2^.

### Cultivation of Derived Cells Postdifferentiation

Exchange ECFC cultivation medium every other day and passage the cells using TrypLE when they reach 90% confluence. In all subsequent passages, seed at a density of 7,000 cells/cm^2^.

### Analysis of Derived Cells

**FIGURE 3:**
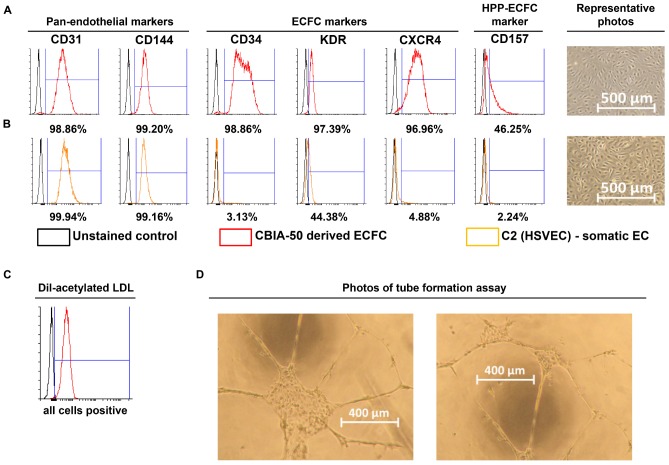
Characterization of cells derived by our synergistic three-phase protocol and their comparison with somatic endothelial cells (ECs). Representative flow cytometry histogram overlays represent the surface expression of CD31, CD144, CD34, KDR, CXCR4, and CD157 and uptake of Dil-labeled and acetylated low-density lipoprotein (Dil-ac-LDL). **(A)** CBIA-50–derived ECFCs by the end of the first passage; ECFCs were obtained by passaging CBIA-50–derived cells, analyzed in Figure 2D, by day 5 of differentiation without separation. **(B)** Human saphenous vein EC cell line C2 by the end of passage 7; this cell line was harvested and characterized in our laboratory. **(A,B)** Morphology of confluent cells shows similar phenotype in both cell lineages shown. Both cell lines have similar CD31 expression but the CBIA-50–derived cells have much higher expression of markers CD34, KDR, CXCR4, and CD157 than the HSVEC line C2. **(C)** Uptake of Dil-ac-LDL by CBIA-50–derived cells in passage 3 was analyzed by flow cytometry, and the entire population was positive. **(D)** CBIA-50–derived cells formed tubes on Matrigel.

Analyze derived cells by FACS after the first passage in order to verify the purity and quality of the cells. Use markers CD31 and/or CD144 to analyze purity and CD34, KDR, CXCR4, and CD157 to determine the ECFC character of the cells (Figure 3A). After three passages, the endothelial character of the derived cells can be further verified by measuring low-density lipoprotein (LDL) uptake and by conducting a tube formation assay, as described below.

Incubate the derived cells with 10 mg/mL Dil-Ac-LDL for 4 h and then harvest them as a single-cell suspension using TrypLE and resuspend them in 300 μL PBS containing 0.5% BSA and 2 mM EDTA. Analyze this suspension by FACS (Figure 3C).

Coat a 96-well microplate for angiogenesis (Ibidi, Planegg, Germany) with 25 μL/well Matrigel and incubate it at 37°C for 1 h. Seed the derived cells at densities of 5,000, 10,000, and 15,000 cells/well in endothelial cell growth medium 2 supplemented with 50 ng/mL VEGF_165_ (50 μL/well) and incubate in a 37°C incubator with a 5% CO_2_ atmosphere for 24 h to allow tubes to form. Analyze tube formation by light microscopy (Figure 3D).

### Additional Information

All media exchanges in the experiment were conducted at room temperature. All cells were cultured in an incubator at 37°C with a high humidity and a 5% CO_2_ atmosphere. Representative images of predifferentiation hPSCs, the entire differentiation process, and postdifferentiation derived cells can be seen in Figure 2.

### Notes on FACS Analysis

Resuspend pellet of single cells in PBS containing 0.5% BSA and 2 mM EDTA. Use 100 μL of PBS/BSA/EDTA mixture per sample. Use at least 30,000 living cells per sample in order to get at least 10,000 relevant events during your FACS analysis. Combine two different types of antibodies (APC-conjugated + PE-conjugated antibodies) per sample if possible. Incubate the mixture with fluorochrome-conjugated antibodies for 15 min at 4°C. You can either use the manufacturer-recommended amount of antibodies or a reduced amount, for example, 3 to 4 μL instead of 10 μL or 1 μL instead of 2 μL. This reduction of antibody volume is possible because manufacturer-recommended amounts of antibodies are set for 1 million or more cells per sample, whereas a much smaller sample is sufficient. After incubation with antibodies, add 1 mL of refrigerated PBS per sample and centrifuge at 300 × *g* for 3 min at 4°C. Remove the supernatant and add 300 μL of refrigerated PBS to the pellet. Ideally, analyze it as soon as possible. If necessary, samples can last in this state for up to 2 h. Pipet or vortex the mixture for a few seconds prior to analysis.

### Notes on MACS Analysis

Resuspend pellet of single cells in PBS containing 0.5% BSA and 2 mM EDTA. The same PBS/BSA/EDTA mixture can be used for FACS and MACS. For example, prepare 1 mL of the PBS/BSA/EDTA mixture and then use 3 × 100 μL of it for FACS (for the unstained control, anti–CD31-APC + anti–CD34-PE and anti–CD144-APC + anti–KDR-PE experiments) and 700 μL of it for MACS. From this point on, follow the MACS instructions of the manufacturer. Skip MACS when either CD31 or CD144 expression is ≥85% (Figure 2D).

### Advice

In addition to your main sample(s), seed one or two additional small format dishes, for example, 12-well size. Use cells from these 12-well dishes for FACS analysis by the end of days 2 and 5. In our experience, the efficiency of differentiation is dependent on the cell line of source hPSCs (Supplementary Figures S1–S3). Variability between different samples of the same hPSC line is negligible. Therefore, the results of FACS analysis from a smaller sample can be used to determine whether MACS is necessary for a larger sample.

## Expected Results

**FIGURE 4:**
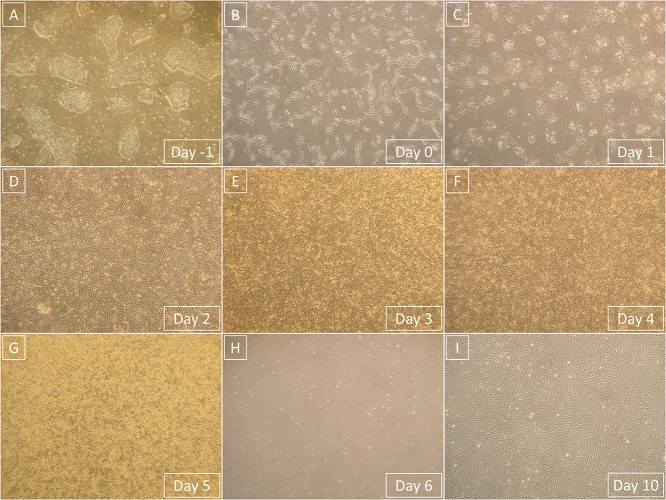
Representative light microscopy photographs that show the entire process of differentiation from hPSCs to derived ECFCs. Magnification in all images is 40×. **(A)** hPSCs prior to single-cell seeding. **(B)** hPSCs 1 day after the single-cell seeding. **(C)** Primitive streak that formed from hPSCs after 1-day exposure to phase 1 medium. **(D)** Mesoderm that formed from primitive streak after 1-day exposure to phase 2 medium. **(E)** Endothelium that started to form from mesoderm after 1-day exposure to phase 3 medium. **(F)** Endothelium continuously formed after 2-day exposure to phase 3 medium. **(G)** Endothelium is fully formed and ready to be processed after 3-day exposure to phase 3 medium. **(H)** Derived ECFCs at the beginning of the first passage 1 day after seeding. **(I)** Confluent-derived ECFCs by the end of the first passage; confluence was mostly achieved 4 days after initial seeding.

We employed previously identified effectors of differentiation (Tan et al., 2013; Sahara et al., 2014; Patsch et al., 2015; Sriram et al., 2015; Kempf et al., 2016) into a synergistic three-phase differentiation protocol (Figure 1). With this synergistic approach, we were able to differentiate hPSCs into mesoderm with up to 93% surface expression of KDR and low expression of CXCR4 by day 2 (Figure 2A). During the mesoderm–endothelial transition, the expression of markers CD34, CD31, and CD144 progressively increased. Specifically, on day 3, some cells were strongly expressing CD34 and dimly coexpressing CD31 in addition to overall expression of KDR (Figure 2B). On day 4, approximately 80% of all cells expressed CD31, CD34, and CD144 on their surface in addition to overall expression of KDR (Figure 2C). Finally, by the end of differentiation on day 5, endothelium with up to 97% surface expression of endothelial markers was present (Figure 2D). By the end of the first passage, differentiated ECFCs had high surface expression of the ECFC markers CXCR4, CD34, and KDR and the HPP-ECFC marker CD157 in addition to the standard pan-endothelial surface markers CD31 and CD144 (Figure 3A). We compared derived ECFCs to the somatic HSVEC line C2 derived in our laboratory. Expression of pan-endothelial markers was similar in both cell types, but expression of all the ECFC markers was much lower in the HSVECs than in the derived ECFCs (Figure 3B). CXCR4, CD34, and KDR are often highly expressed on the surface of ECFCs (Joo et al., 2015; Kang et al., 2015), which highlights the ECFC character of our hPSC-derived cells. Even more interestingly, it was recently discovered that CD157 is present on the surface of all HPP-ECFCs (a rare subpopulation of ECFCs), but it was missing on standard ECFCs and mature ECs *in vivo* (Wakabayashi et al., 2018). High expression of CD157 on the surface of endothelium derived using our protocol therefore suggests the HPP-ECFC character of these cells. Additionally, our cells had cobblestone morphology when observed by light microscopy (Figures 3A, 4I), the entirety of their population took up Dil-Ac-LDL (Figure 3C), and they formed tubes on Matrigel (Figure 3D).

**FIGURE 5:**
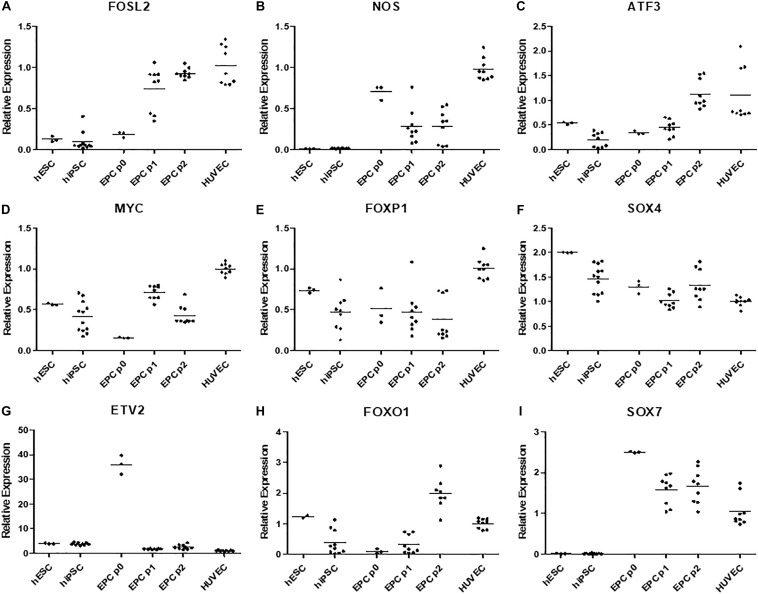
**(A)** Gene FOSL2 has low expression in both hPSC types, its expression is higher in p0 derived cells and peaks in later passages of these cells and HUVECs. **(B)** Gene NOS have negligible expression in hPSCs, it is expressed in derived cells, however highest expression is recorded in HUVECs. **(C)** Gene ATF3 has low expression in hPSCs and passage 0–1 derived cells, its expression is high in passage 2 of derived endothelium and in HUVECs. **(D)** Gene MYC is moderately expressed hPSCs and passage 1 and 2 of derived cells, surprisingly its expression is low in passage 0 of derived cells and highes in HUVECs. **(E)** Gene FOXP1 was highly expressed in HUVECs and moderately expressed in all other cell types. **(F)** Expression of SOX4 gradually decreased from hPSCs to derived cells and HUVECs. **(G)** Gene ETV2 had the lowest expression in HUVECS multiple times higher expression in all other cell types and order of magnitude higher expression in p0 derived cells. **(H)** FOXO1 was moderately expressed in hESCs and HUVECs, highly expressed in p2 derived cells and it had low expression in hiPSCs and p0 and p1 derived cells. **(I)** SOX7 had negligible expression in all hPSCs high expression in p0 derived cells, gradually lower expression in following passages 1 and two and moderate expression in HUVECs.

Intrigued by this, we decided to compare gene expression in our derived cells in passages 0 (cells by the day 5 of differentiation), 1, and 2 to hPSCs and HUVECs via quantitative polymerase chain reaction (Figure 5). We chose some of the genes that were previously linked to CD157-positive ECFCs (Wakabayashi et al., 2018), specifically FOXO1, FOXP1, MYC, FOSL2, ATF3, SOX4, and SOX7. In addition, we measured expression of ETV2, which can induce endothelial differentiation on its own, and NOS, which is marker of mature endothelial functionality. Expression of FOXP1, MYC, FOSL2, ATF3, and NOS gradually increased from pluripotent stem cells, through derived cells to mature HUVEC (Figures 5A–E). Expression of SOX4, a marker of pluripotency, gradually decreased from hPSCs, through derived cells to mature HUVECs (Figure 5F). ETV2 was more expressed in both hPSCs and derived cells than in HUVECs; interestingly, at passage 0, it was overexpressed by an order of magnitude in comparison to other passages of the derived cells and pluripotent stem cells (Figure 5G). FOXO1 had higher expression in derived cells by passage 2 than in all other tested cells (Figure 5H). Finally, SOX7 was more expressed in all derived cells than in pluripotent stem cells and HUVECs (Figure 5I).

**FIGURE 6:**
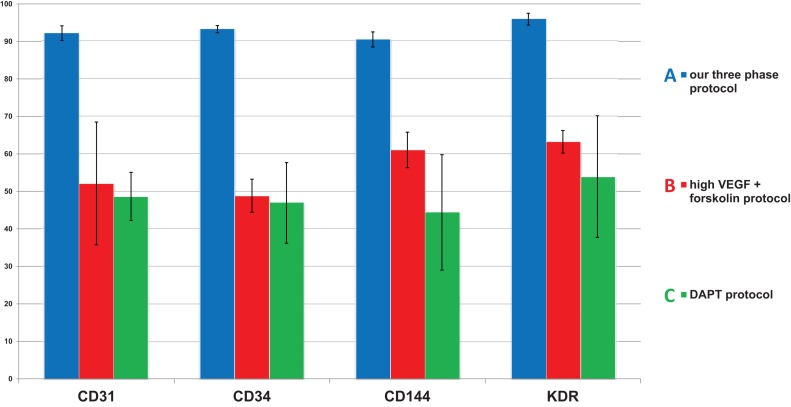
Comparison of mean endothelial surface marker expression levels between our synergistic three-phase protocol and two N2B27 + StemPro protocols (high VEGF + forskolin protocol and DAPT protocol). Mean and standard deviation values were obtained from biological duplicates of differentiation experiments using CBIA-37 and CBIA-50 cell lines on day 5 of differentiation. Analysis was conducted via flow cytometry. The mean expression of surface markers CD31, CD144, CD34, and KDR was **(A)** 90% to 96% for our protocol, **(B)** 49% to 63% for the high VEGF + forskolin protocol, and **(C)** 44% to 54% for the DAPT protocol. Variability in the surface marker expression was lower for our protocol than the other tested protocols, as shown by the standard deviations.

We decided to compare the efficiency and robustness of our protocol with two previously published protocols that involve only some of the differentiation effectors used in our protocol (Sahara et al., 2014; Patsch et al., 2015). Differences between our protocol and these two protocols are detailed in Table 1. Briefly these two protocols involve two phases of differentiation: (i) hPSC to mesoderm and (ii) mesoderm to endothelium. They both utilize N-2 Supplement (100×) + B-27 Supplement (50×) along with either Gibco^TM^ Neurobasal^TM^ Medium (Thermo Fisher Scientific) and DMEM2 (Patsch et al., 2015) or only DMEM2 (Sahara et al., 2014) in phase 1 of differentiation and StemPro-34 serum-free medium (SFM; 1×) (Thermo Fisher Scientific) in phase 2 (i.e., two variants hereafter referred to as N2B27 + StemPro). They mainly differ from one another in their phase 2 usage of 2 μM forskolin with a high dosage of 200 ng/mL VEGF_165_ (Patsch et al., 2015) or 10 μM DAPT with a more conservative dose of 50 ng/mL VEGF_165_ (Sahara et al., 2014), respectively. Efficiency of differentiation was measured by FACS analysis of cell cultures on day 5 of differentiation. Our synergistic three-phase protocol had >90% expression of the markers CD31, CD34, CD144, and KDR (Figure 6A). The high VEGF + forskolin protocol had 36 to 69% expression for markers CD31, CD34, CD144, and KDR (Figure 6B). The DAPT protocol had 27 to 68% expression for markers CD31, CD34, CD144, and KDR (Figure 6C). For both the high VEGF + forskolin protocol and the DAPT protocol, most of the deviation was between different cell lines used; there was only a small deviation between pairs of samples of the same cell line (Supplementary Figures S1–S3). We concluded that the efficiency of these two protocols is generally lower and more cell line–dependent than the efficiency of our synergistic protocol. Additionally, there was heterogeneity in the differentiation process itself when N2B27 + StemPro media were used. Sometimes, when N2B27 medium was exchanged for StemPro medium (Supplementary Figure S6), the entire monolayer cell population lost adherence to the surface only to adhere back to the surface later as a sphere-shaped structure. Endothelial cells then sprouted from this sphere-shaped structure in a root-like manner (Supplementary Figure S6). In some cases, this did not happen at all; instead, some parts of the monolayer mesoderm population eventually transformed into endothelium. However, when our synergistic three-phase protocol, which employs STEMdiff APEL2 medium, was used, cells grew in a monolayer, and the vast majority of them reliably turned into endothelium in an orderly and predictable manner (Figures 4B–G). Finally, certain elements of the differentiation process (such as the volume of the medium or whether the differentiation medium should be refreshed daily) were not fully described in the articles describing the two previous N2B27 + StemPro protocols (Sahara et al., 2014; Patsch et al., 2015). In contrast, we are describing each step of differentiation in full detail, which should help with the adaptation of our protocol in different laboratories.

**TABLE 1:** Description of basic properties of our synergistic three-phase differentiation protocol and protocols developed by Patsch et al. (2015) (nicknamed high VEGF + forskolin protocol in our article) and Sahara et al. (2014) (nicknamed DAPT protocol in our article).

**Protocol**	**Duration of differentiation**	**Primitive streak and mesoderm differentiated by separate media (precision of differentiation)**	**Derived cells tested for standard surface markers**	**Cytokines used in each stage of differentiation: primitive streak = PS, mesoderm = M, endothelium = E**	**Media used in each stage of differentiation: primitive streak = PS, mesoderm = M, endothelium = E**	**Number of hPSC lines tested (robustness)**	**Average percentage of successfully differentiated cells (efficiency)**	**Precise single cell seeding density/media volumes defined (reproducibility and standardization)**
Our synergistic three phase protocol (Farkas et al.)	5 days	Yes	CD31, CD34, CD144, KDR	PS: CP21R7, BMP4, FGF2 M: BMP4, FGF2 E: high concentration VEGF165, DAPT, Forskolin	PS: STEMdiff APEL2 M: STEMdiff APEL2 E: STEMdiff APEL2	7	85–94% for CD31, CD34, CD144, KDR	Yes – 400 000 cells per pm35 well*/yes – 1 ml per pm35 well in stage 1, 2 ml per well in stage 2 and three
Patsch et al., 2015 (original article results)	5 days	No	CD31, CD144, vWF	M: BMP4, CP21R7 E: high concentration VEGF165, Forskolin	M: N2B27 + Neurobasal medium E: StemPro34	4	70.1 % CD144	370000–470000/No
Patsch et al., 2015 (our test results)	5 days	No	CD31, CD34, CD144, KDR	M: BMP4, CP21R7 E: high concentration VEGF165, Forskolin	M: N2B27 + Neurobasal medium E: StemPro34	2	49–61% for CD31, CD34, CD144, KDR	Yes – 400 000 cells per pm35 well */yes 3 ml per pm35 well in each stage, no refreshment of medium – most efficient method we tested
Sahara et al., 2014 (original article results)	5 days	No	CD31, CD34, CD144, KDR	M: BMP4, CP21R7 E: VEGF165, DAPT	M: N2B27 + Neurobasal medium + DMEM-F12 E: StemPro34	4	50% CD31 + CD144	No/No
Sahara et al., 2014 (our test results)	5 days	No	CD31, CD34, CD144, KDR	M: BMP4, CP21R7 E:VEGF165, DAPT	M: N2B27 + Neurobasal medium + DMEM-F12 E: StemPro34	2	44–54% for CD31, CD34, CD144, KDR	Yes – 400 000 cells per pm35 well* /yes 3 ml per pm35 well in each stage, no refreshment of medium – most efficient method we tested

Table includes relevant properties such as duration, precision, robustness, efficiency, reproducibility and standardization of differentiation, tested surface markers, cytokines, and media used in each stage of differentiation. Table includes information from both original articles written by their respective authors and our tests separately. *pm35 dish 10 cm^2^.

**FIGURE 7:**
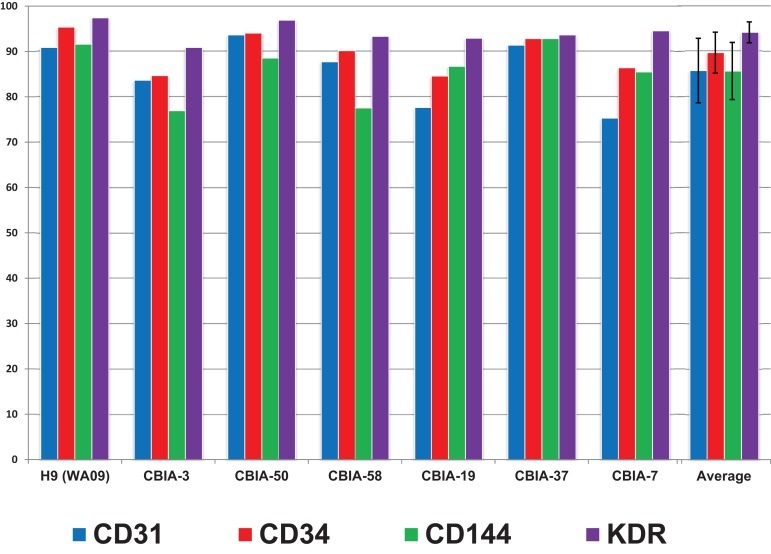
Surface marker expression of cells differentiated by synergistic three-phase protocol from multiple hPSC lines by day 5 of differentiation along with the mean and standard deviation values across all hPSC lines used. The hESC line used was H9 (WA09), and the six hiPSC lines used were CBIA-3, CBIA-50, CBIA-58, CBIA-19, CBIA-37, and CBIA-7. Analysis of surface markers CD31, CD34, CD144, and KDR was conducted via flow cytometry. Expression of these markers ranged between 75% and 97%. Mean expression of these markers was between 85 and 94%.

Furthermore, we tested the robustness of our synergistic three-phase protocol on multiple hPSC lines comprising an hESC line and six hiPSC lines derived using virus, episomal vector, and mRNA induction techniques. Our protocol required no additional individual optimization for specific hPSC lines in order to achieve, on average, high differentiation efficiency (85%–94%) (Figure 7 and Supplementary Figure S4). In addition, the tested hPSC lines tended to achieve high-enough endothelial surface marker expression to entirely exclude the need for cell separation. This considerably increased final yields, as when magnetic separation was applied it resulted in up to 50% loss of cells positive for the selected marker. As a result, the final differentiation efficiency is up to 1,500% (one stem cell gives rise to up to 15 differentiated cells) without using any separation method.

## Troubleshooting

### Potential Problem 1

Expression of both surface markers CD31 and CD144 is <85% on cells by day 5 of differentiation.

### Solutions to Potential Problem 1

Try seeding your hPSCs at higher densities, for example, 45,000 or 50,000/cm^2^. Make sure your hPSCs are above passage 15; if you want to be totally safe, use hPSCs at passage 20 or higher. Cultivate your cells in mTeSR-1 (or a similar commercial medium if you have prior positive experience with it) in dishes coated with Matrigel (or a similar matrix) for at least three passages prior to single-cell seeding for differentiation. Make sure that your hPSCs have a healthy karyotype. Ideally, use hiPSCs below passage 30; do not use hiPSCs above passage 40 unless you are certain they are in perfect condition (related to karyotype, genomic mutations, and epigenetics). If none of this advice works for you, either use MACS to separate the derived cells or try using a different hPSC line.

### Potential Problem 2

Expression of pan-endothelial markers CD31 and CD144 is <97% in derived ECFCs by the end of the first passage.

### Solution to Potential Problem 2

Cultivate the cells for one more passage. If the expression does not increase, use MACS to separate the cells, employing microbeads against either CD31 or CD144.

### Potential Problem 3

Derived ECFCs do not express CD157 on their surface.

### Solution to Potential Problem 3

Try your anti-CD157 antibody against ECFCs derived from a different hPSC line to ensure that the antibody actually works. If the antibody functions correctly, it is likely that the hPSCs used to derive the first ECFCs have some issue with their karyotype.

## Discussion

We created our synergistic protocol by employing multiple effectors of endothelial differentiation that were previously used and studied separately (Tan et al., 2013; Sahara et al., 2014; Patsch et al., 2015; Sriram et al., 2015; Kempf et al., 2016) to reliably generate ECFCs in large numbers. Therefore, our synergistic protocol involves a different medium for each of the three phases of differentiation. A schematic description of the entire differentiation process is shown in Figure 1 and representative photos of entire procedure from pre-differentiation to post-differentiation state are shown in Figures 4A–I. A comparison of our protocol with many protocols cited in this article is shown in Table 2. During phase 1, a primitive streak was induced from hPSCs by a high dosage of a GSK3-β inhibitor in a low volume of medium (Tan et al., 2013; Kempf et al., 2016). The efficiency of this process was further increased by adding the cytokine BMP4, which primes the primitive streak toward KDR^+^ mesoderm (Orlova et al., 2014; Sahara et al., 2014; Patsch et al., 2015; Sriram et al., 2015) and the cytokine FGF2, which increases the proliferation of the forming mesoderm (Sriram et al., 2015). During phase 2, the GSK3-β inhibitor is omitted as prolonged exposure to this inhibitor differentiates the primitive streak into definitive endoderm instead of mesoderm (Tan et al., 2013). Again, BMP4 was used to ensure the differentiation of the primitive streak into mesoderm, whereas FGF2 supported the proliferation of the new mesoderm. During phase 3, mesoderm was differentiated into endothelium by a high dosage of VEGF-A_165_. Forskolin (a positive regulator of cAMP and protein kinase A) was added to the medium in order to maximize the effect of VEGF-A_165_ by increasing the expression of its receptors, Neuropilin 1 and KDR (Yamamizu et al., 2009; Patsch et al., 2015). Finally, the Notch signaling inhibitor DAPT was utilized to promote the proliferation of newly forming ECFCs and simultaneously prevent their maturation during phase 3 of differentiation (Sahara et al., 2014). This procedure resulted in an almost pure population of endothelial progenitors by the end of differentiation (Figures 2D, 6A, 7). After one passage, the derived ECs fitted the profile of HPP-ECFCs when compared to the somatic HSVEC line (Figure 3). Finally, when gene expressions of hPSCs, derived ECs in passages 0 to 2 and HUVECs in passages 3 to 4 were compared, the derived ECs were mostly intermediate between hPSCs and HUVECs in their gene expression. In case of FOSL2, ATF3, MYC, NOS, and FOXP1, their expressions gradually increased from hPSCs to endothelium. SOX4 behaved exactly opposite as its expression gradually decreased from hPSCs to endothelium. There were notable exceptions to this general behavior. Expression of ETV2 that was already elevated in hPSCs spiked by the end of differentiation and then stayed elevated in derived ECs in comparison to HUVECs; this implies key role of ETV2 in endothelial differentiation. Expression of SOX7 was negligible in hPSCs but elevated in all derived ECs in comparison to HUVECs. FOXO1 had lower expression in derived ECs in passages 0 and 1 but elevated expression by passage 2 in comparison to both hPSCs and HUVECs. These results suggest endothelial characteristics of the derived cells but of less mature type than cells such as HUVECs.

**TABLE 2:** Description of basic properties of our synergistic three-phase differentiation protocol and other monolayer differentiation protocols cited in this article.

**Protocol**	**Duration of differentiation**	**Primitive streak and mesoderm differentiated by separate media (precision of differentiation)**	**Derived cells tested for standard surface markers**	**Derived cells tested for advanced progenitor markers or properties**	**Derived cells tested by tube forming assay/LDL uptake**	**Number of hPSC lines tested (robustness)**	**Percentage of successfully differentiated cells/derived cells yield per pm35 (efficiency)**	**Precise single cell seeding density/media volumes defined (reproducibility and standardization)**	**Cytokines or media used require individualized dosage for different PSC lines (reproducibility and standardization)**
Our synergistic three phase protocol (Farkas et al.)	5 days	Yes	CD31, CD34, CD144, KDR	Surface markers CXCR4, CD157	Yes/Yes	7	85–94% CD31, CD34, CD144, KDR/up to 6 million cells	Yes/Yes	No
Patsch et al., 2015	5 days	No	CD31, CD144, vWF	*In vivo* test on mice, transcriptome	Yes/Yes	4	70.1% CD144/up to 8 million cells	No/No	No
Sahara et al., 2014	5 days	No	CD31, CD34, CD144, KDR	PCR array, lack of CD14, single cell assay, proliferation assay	Yes/No	4	50% CD31 + CD144	No/No	No
Sriram et al., 2015	5 days	Yes	CD31, CD34, KDR	Surface markers CXCR4, NRP1, migration assay, *in vivo* test in mice	Yes/Yes	2 (only hESC, no hiPSC)	90–95% CD31, CD34, KDR	No/No	No
Prasain et al., 2014	12 days	No	CD31, CD144, KDR	Surface marker NRP1, colony forming test, *in vivo* test on mice	Yes/No	4	Illegible but sorting necessary/3750 cells	No/Yes	No
Park et al., 2010	10–15 days + sorting +12 days	No	CD31, CD34, CD105	Colony forming, *in vivo* test on mice	Yes/Yes	2	10–16% CD31 + CD34/not mentioned	No/No	Yes
Harding et al., 2017	4–10 days	No	CD31, CD144, CD34 (negative)	No	Yes/Yes	2	67.8% CD31+CD144-day 4/further manual separation	No/No	Yes
Orlova et al., 2014	10–11 days	No	CD31, CD105, CD73, CD144, KDR	*In vivo* test in zebra fish, gene expression	Yes/No	3	19.9%/not mentioned	No/Yes	No
Tatsumi et al., 2011	5 days	No	CD31, CD34, CD144, KDR	No	Yes/Yes	4	20%/1.2 million cells	No/No	No
Bao et al., 2015	5 days	No	CD31, CD34, CD144, vWF	No	Yes/Yes	6	24.45 %CD31, CD34/not mentioned	No/No	Yes

*Table includes relevant properties such as duration, precision, robustness, efficiency, reproducibility and standardization of differentiation, tested surface markers, tested progenitor properties, LDL uptake, and tube assay. Table includes information from original articles written by their respective authors.*

Interestingly, when phase 2 (mesoderm) of our differentiation process was prolonged by 1 day, KDR expression was high and similar on both days 2 (Figure 3E) and 3 (Figure 4F). However, this did not translate into increased final endothelial differentiation efficiency. Surprisingly, by day 5 of differentiation, the expression of the endothelial markers CD31, CD34, and CD144 was significantly decreased (Figure 3G) in comparison to the standard version of our protocol (Figure 3D) involving only 1 day of phase 2. To explain this behavior, we hypothesize that mesoderm needs to be driven toward endothelium very soon after its appearance in order to avoid spontaneous differentiation toward different cell types. Additionally, it seems that the potential for endothelial differentiation precedes the actual appearance of mesoderm with KDR on its surface. In other words, the majority of cells (including KDR^–^ cells of the primitive streak) are sufficiently primed toward mesoderm by day 2 of differentiation when our medium is used. Therefore, the phase 3 (endothelial differentiation) medium cannot disturb the eventual differentiation of these cells toward KDR^+^ mesoderm. During phase 3, already present KDR^+^ mesoderm differentiates to endothelial progenitors, whereas KDR^–^ cells mature into KDR^+^ mesoderm and then follow the aforementioned fate.

Next, we compared the synergistic effect of our protocol with the effects of two other previously published protocols (Sahara et al., 2014; Patsch et al., 2015) that use only some of the compounds and cytokines used in our protocol. We found that the efficiency of differentiation was higher for our protocol (Figure 6A) in comparison to the other two protocols (Figures 6B,C). Additionally, the variability in differentiation efficiency between two different hPSC lines was lower with our protocol than with the other two protocols (Figure 6). Variability in differentiation efficiency between pairs of samples from the same hPSC line was very low for all protocols used (Supplementary Figures S1–S3). Additionally, cells differentiated by our protocol behaved in a very orderly and predictable manner during the differentiation process (Figures 4B–G). Surprisingly, all N2B27 + StemPro protocols that we tested behaved rather unpredictably during the mesoderm–endothelium transition. Specifically, sometimes when N2B27 medium was exchanged for StemPro medium, all the cells lost adherence to surface only to adhere later as one big clump (Supplementary Figures S5, S6). We actually tested a version of our protocol that used N2B27 and StemPro instead of STEMdiff APEL2 medium (data not shown), and while the differentiation efficiency was higher than that of the other two N2B27 + StemPro protocols (Sahara et al., 2014; Patsch et al., 2015), the same issues with variability of differentiation efficiency and adherence were present for all three protocols. Because of this, we believe the issues with adherence and variability are mostly due to transition from N2B27 medium to StemPro medium, not due to differences in the small compounds and cytokines. Therefore, we assume that our synergistic three-phase protocol results in predictable behavior and low variability due to the use of STEMdiff APEL2 medium, whereas the high efficiency of differentiation is the result of synergy between the timing of each step, the cytokines, and the small compounds.

In conclusion, our synergistic three-phase protocol differentiates hPSCs into ECFC-like cells via KDR^+^ mesoderm, with higher efficiency, reproducibility, and robustness than other tested protocols. We proved this by successful differentiation of the hESC H9 line and six hiPSC lines derived using the three most common induction techniques (virus, episomal vector, and mRNA). Finally, the differentiated cells expressed the HPP-ECFC surface marker CD157 in addition to the standard pan-endothelial and ECFC markers. Therefore, our protocol is a promising asset in the effort to develop standardized regenerative medicine.

## Data Availability Statement

All datasets generated for this study are included in the article/Supplementary Material.

## Author Contributions

SF co-conceptualized the study, designed the differentiation protocol, drafted the manuscript, performed all cell culture, data collection, analysis and interpretation of results related to the differentiation protocol and following progenitor cultivation, and approved the final manuscript. PS was responsible for derivation, analysis and quality control of hiPSC lines CBIA-3, CBIA-7, CBIA-19, and CBIA-37 used in the differentiation protocol, provision of editorial support, and approval of the final manuscript. DR was responsible for derivation, analysis and quality control of hiPSC lines CBIA-50 and CBIA-58 used in the differentiation protocol, provision of editorial support, and approval of the final manuscript. LV provided tissue necessary for primary cell culture cultivation, further derivation of hiPSC lines from the cell cultures, provision of editorial support, and approved the final manuscript. IK co-conceptualized and supervised the study, provided editorial support, and approved the final manuscript.

## Conflict of Interest

The authors declare that the research was conducted in the absence of any commercial or financial relationships that could be construed as a potential conflict of interest.
