# Supplementary material for: Endothelial Progenitor Cells Produced From Human Pluripotent Stem Cells by a Synergistic Combination of Cytokines, Small Compounds, and Serum-Free Medium
Source: Front Cell Dev Biol. 2020 May 15;8:309. doi: 10.3389/fcell.2020.00309 (PMC7249886; doi:10.3389/fcell.2020.00309)
Supplement: Supplementary file 1 [file Presentation_1.PPTX]

## Slide 1
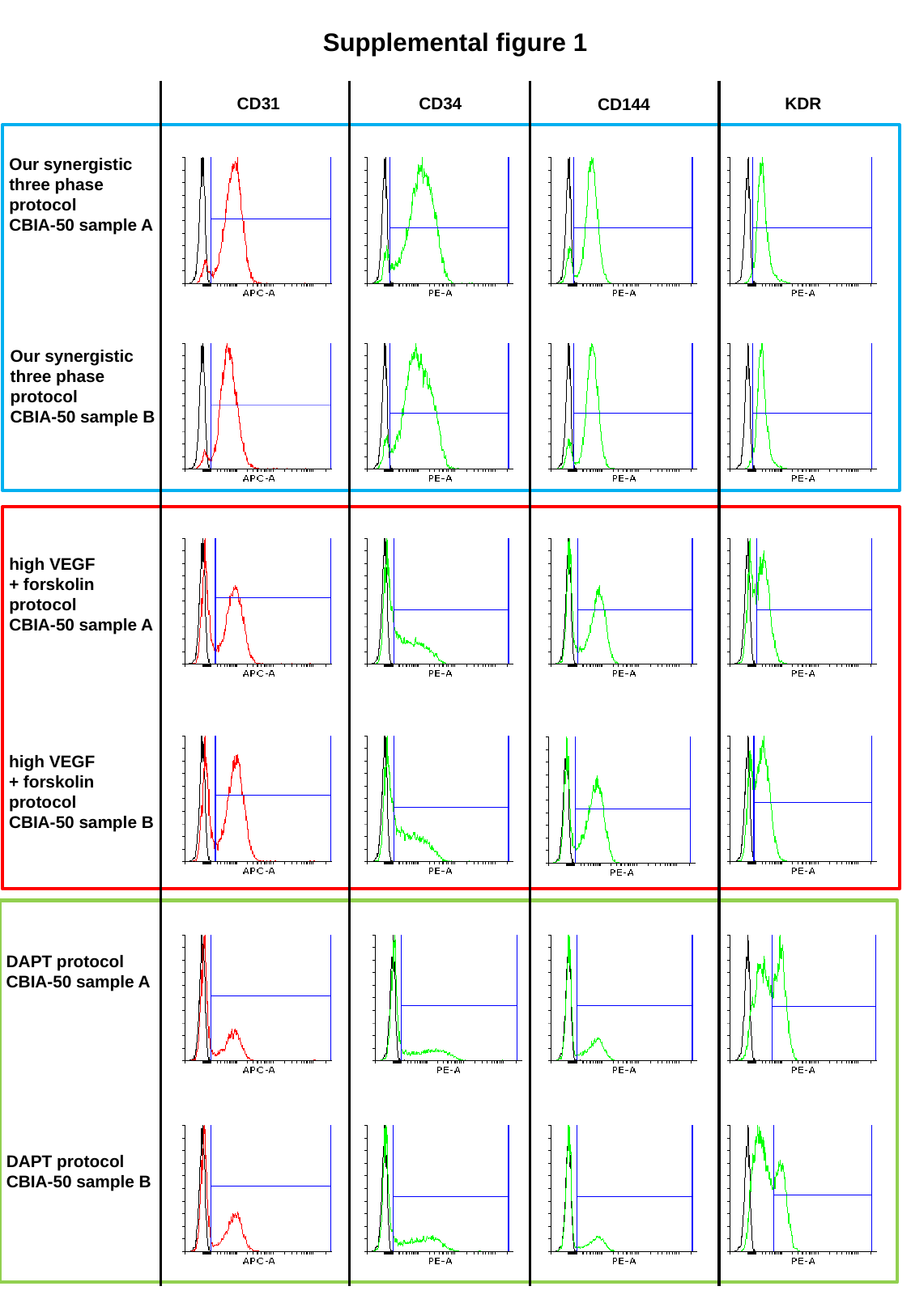

Supplemental figure 1
CD31
CD34
KDR
CD144
Our synergistic three phase protocol
CBIA-50 sample A
Our synergistic three phase protocol
CBIA-50 sample B
high VEGF
+ forskolin protocol
CBIA-50 sample A
high VEGF
+ forskolin protocol
CBIA-50 sample B
DAPT protocol
CBIA-50 sample A
DAPT protocol
CBIA-50 sample B

## Slide 2
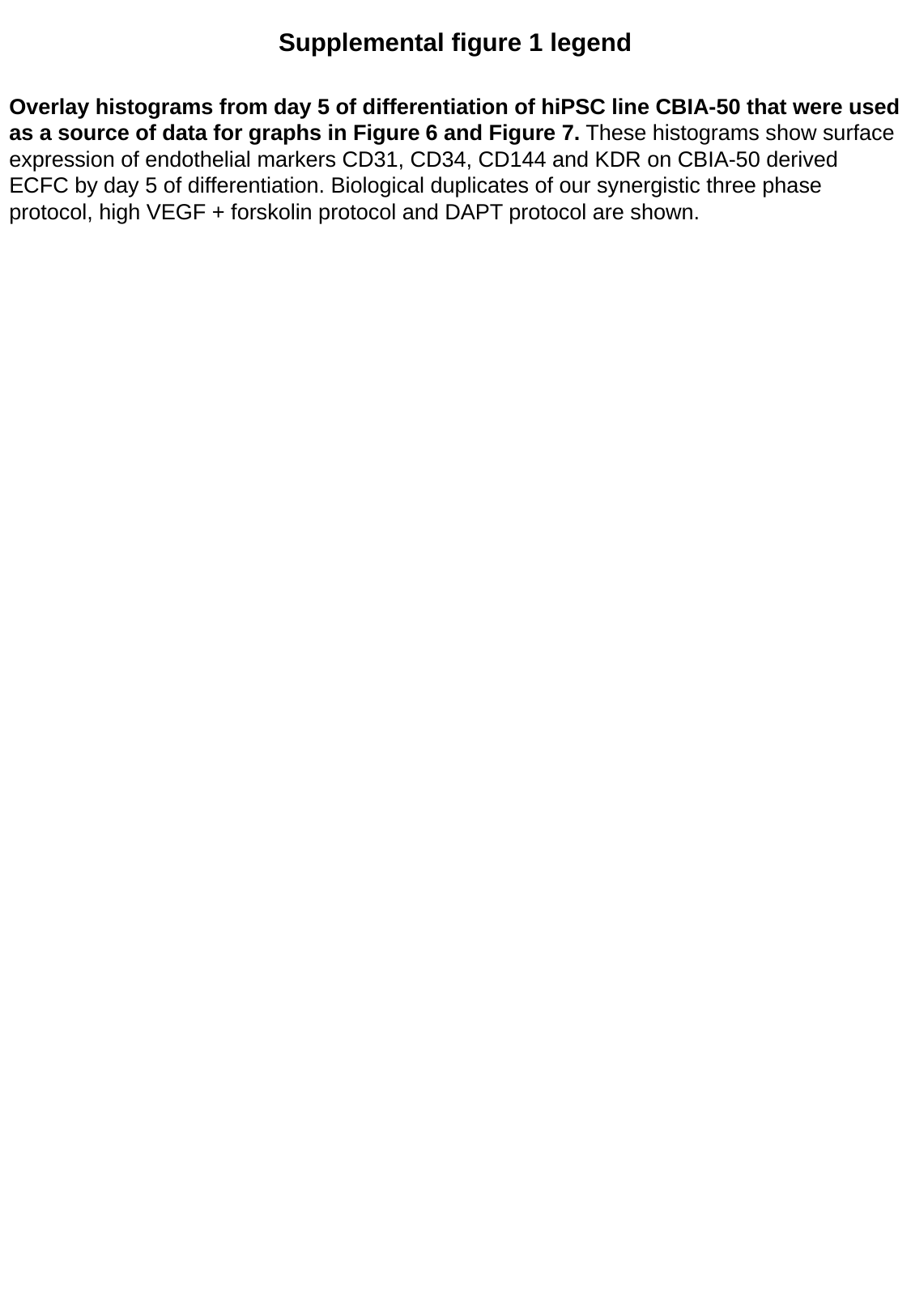

Supplemental figure 1 legend
Overlay histograms from day 5 of differentiation of hiPSC line CBIA-50 that were used as a source of data for graphs in Figure 6 and Figure 7. These histograms show surface expression of endothelial markers CD31, CD34, CD144 and KDR on CBIA-50 derived ECFC by day 5 of differentiation. Biological duplicates of our synergistic three phase protocol, high VEGF + forskolin protocol and DAPT protocol are shown.

## Slide 3
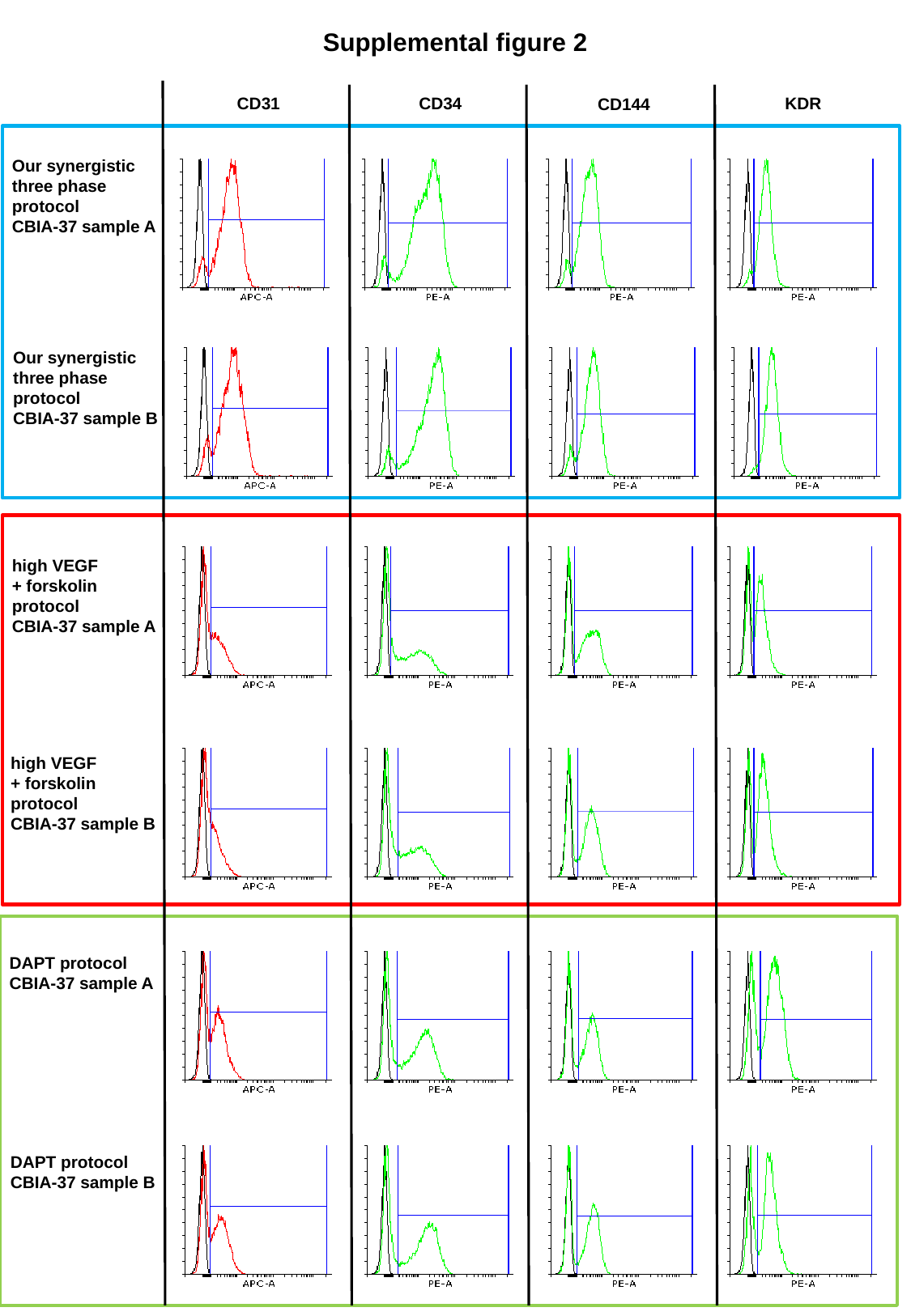

Supplemental figure 2
CD31
CD34
KDR
CD144
Our synergistic three phase protocol
CBIA-37 sample A
Our synergistic three phase protocol
CBIA-37 sample B
high VEGF
+ forskolin protocol
CBIA-37 sample A
high VEGF
+ forskolin protocol
CBIA-37 sample B
DAPT protocol
CBIA-37 sample A
DAPT protocol
CBIA-37 sample B

## Slide 4
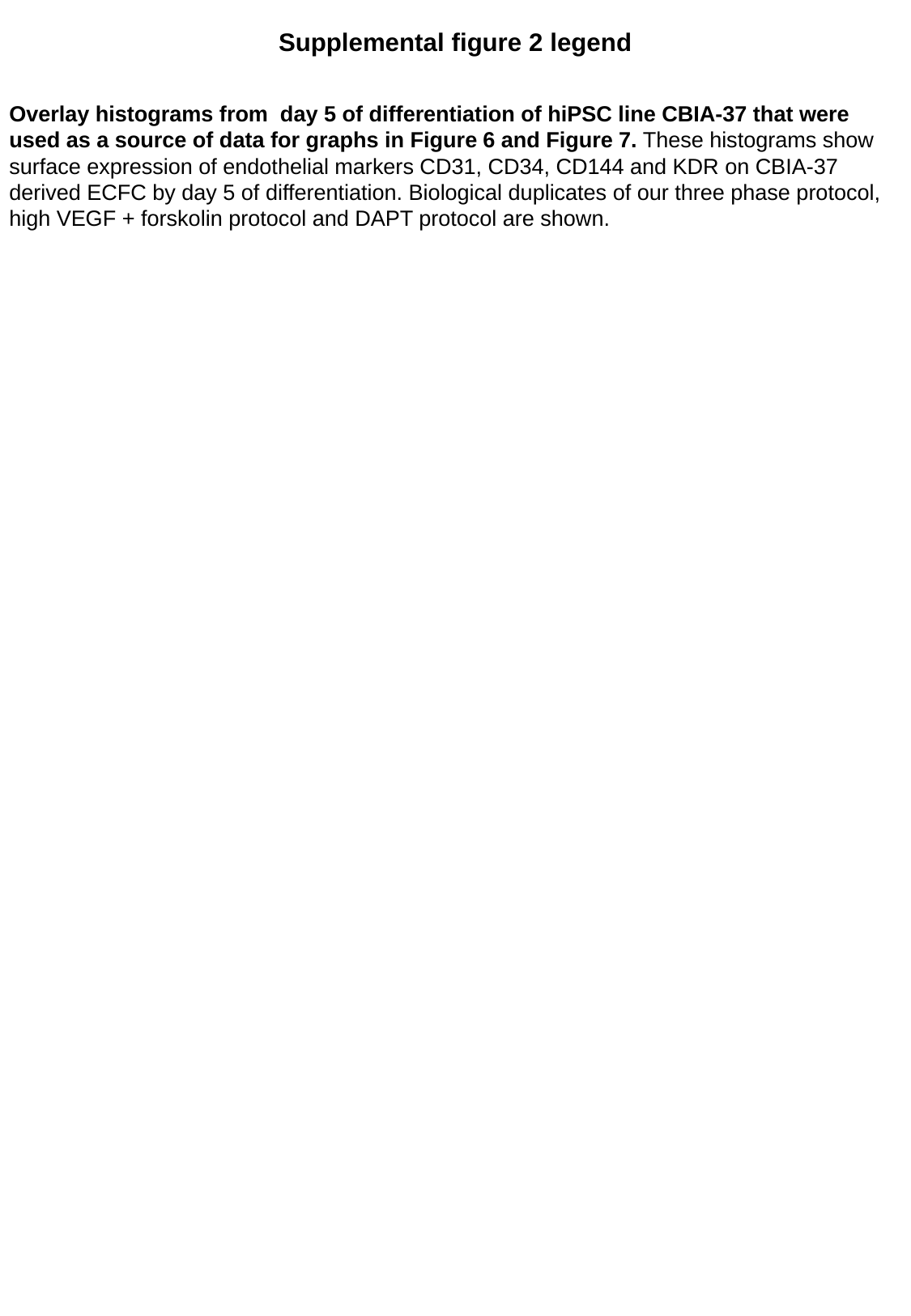

Supplemental figure 2 legend
Overlay histograms from day 5 of differentiation of hiPSC line CBIA-37 that were used as a source of data for graphs in Figure 6 and Figure 7. These histograms show surface expression of endothelial markers CD31, CD34, CD144 and KDR on CBIA-37 derived ECFC by day 5 of differentiation. Biological duplicates of our three phase protocol, high VEGF + forskolin protocol and DAPT protocol are shown.

## Slide 5
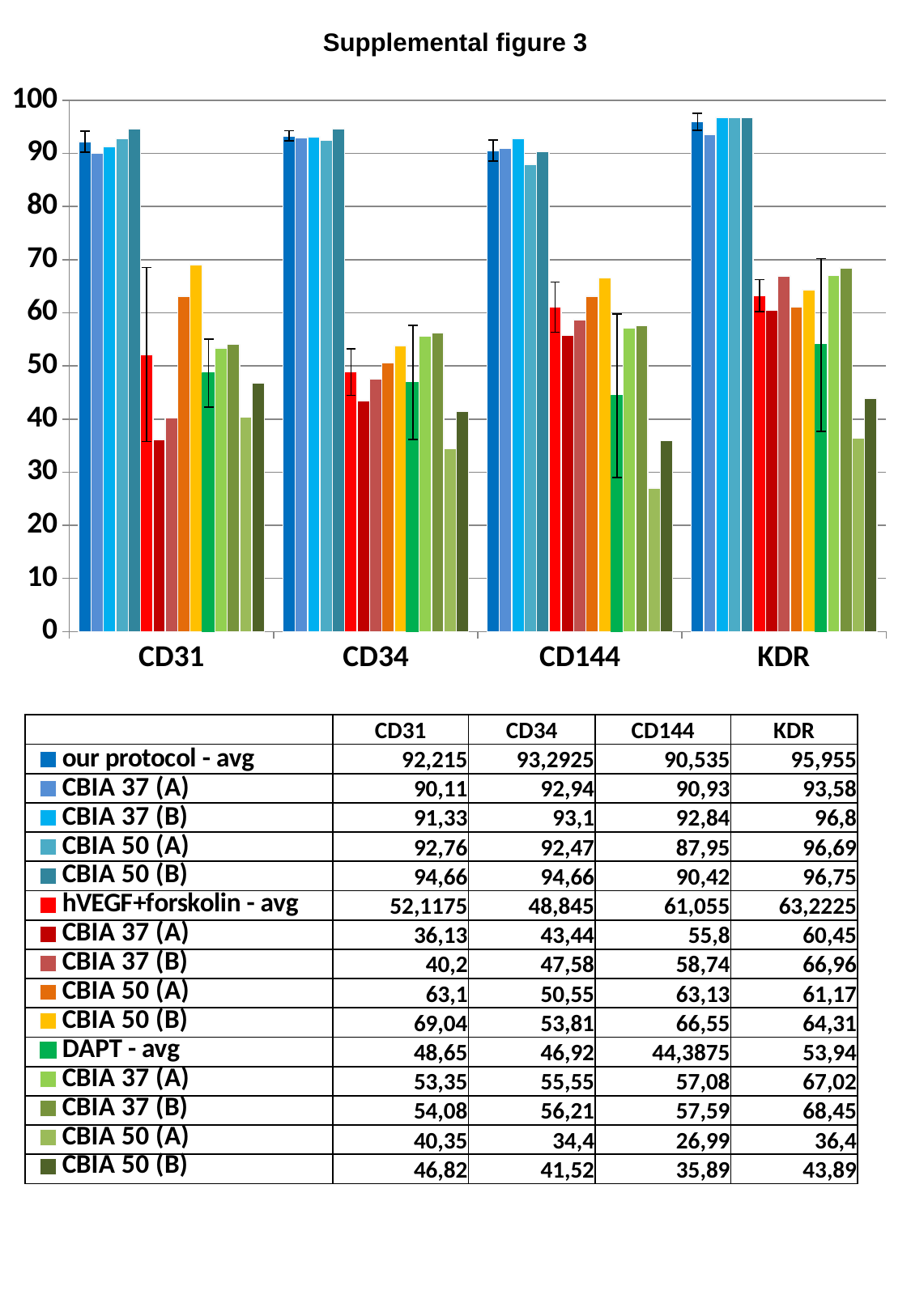

Supplemental figure 3
### Chart
| Category | our protocol - avg | CBIA 37 (A) | CBIA 37 (B) | CBIA 50 (A) | CBIA 50 (B) | hVEGF+forskolin - avg | CBIA 37 (A) | CBIA 37 (B) | CBIA 50 (A) | CBIA 50 (B) | DAPT - avg | CBIA 37 (A) | CBIA 37 (B) | CBIA 50 (A) | CBIA 50 (B) |
|---|---|---|---|---|---|---|---|---|---|---|---|---|---|---|---|
| CD31 | 92.215 | 90.11 | 91.33 | 92.76 | 94.66 | 52.11750000000001 | 36.13 | 40.2 | 63.1 | 69.04 | 48.65 | 53.35 | 54.08 | 40.35 | 46.82 |
| CD34 | 93.29249999999999 | 92.94000000000003 | 93.1 | 92.47 | 94.66 | 48.845 | 43.44 | 47.58 | 50.55 | 53.81 | 46.92 | 55.55 | 56.21 | 34.4 | 41.52 |
| CD144 | 90.53500000000001 | 90.93 | 92.84 | 87.95 | 90.42 | 61.05499999999999 | 55.8 | 58.74 | 63.13 | 66.55 | 44.387499999999996 | 57.08 | 57.59 | 26.99 | 35.89 |
| KDR | 95.95500000000001 | 93.58 | 96.8 | 96.69 | 96.75 | 63.22250000000004 | 60.45 | 66.96 | 61.17 | 64.31 | 53.94 | 67.02 | 68.45 | 36.4 | 43.89 || | CD31 | CD34 | CD144 | KDR |
| --- | --- | --- | --- | --- |
| | 92,215 | 93,2925 | 90,535 | 95,955 |
| | 90,11 | 92,94 | 90,93 | 93,58 |
| | 91,33 | 93,1 | 92,84 | 96,8 |
| | 92,76 | 92,47 | 87,95 | 96,69 |
| | 94,66 | 94,66 | 90,42 | 96,75 |
| | 52,1175 | 48,845 | 61,055 | 63,2225 |
| | 36,13 | 43,44 | 55,8 | 60,45 |
| | 40,2 | 47,58 | 58,74 | 66,96 |
| | 63,1 | 50,55 | 63,13 | 61,17 |
| | 69,04 | 53,81 | 66,55 | 64,31 |
| | 48,65 | 46,92 | 44,3875 | 53,94 |
| | 53,35 | 55,55 | 57,08 | 67,02 |
| | 54,08 | 56,21 | 57,59 | 68,45 |
| | 40,35 | 34,4 | 26,99 | 36,4 |
| | 46,82 | 41,52 | 35,89 | 43,89 |

## Slide 6
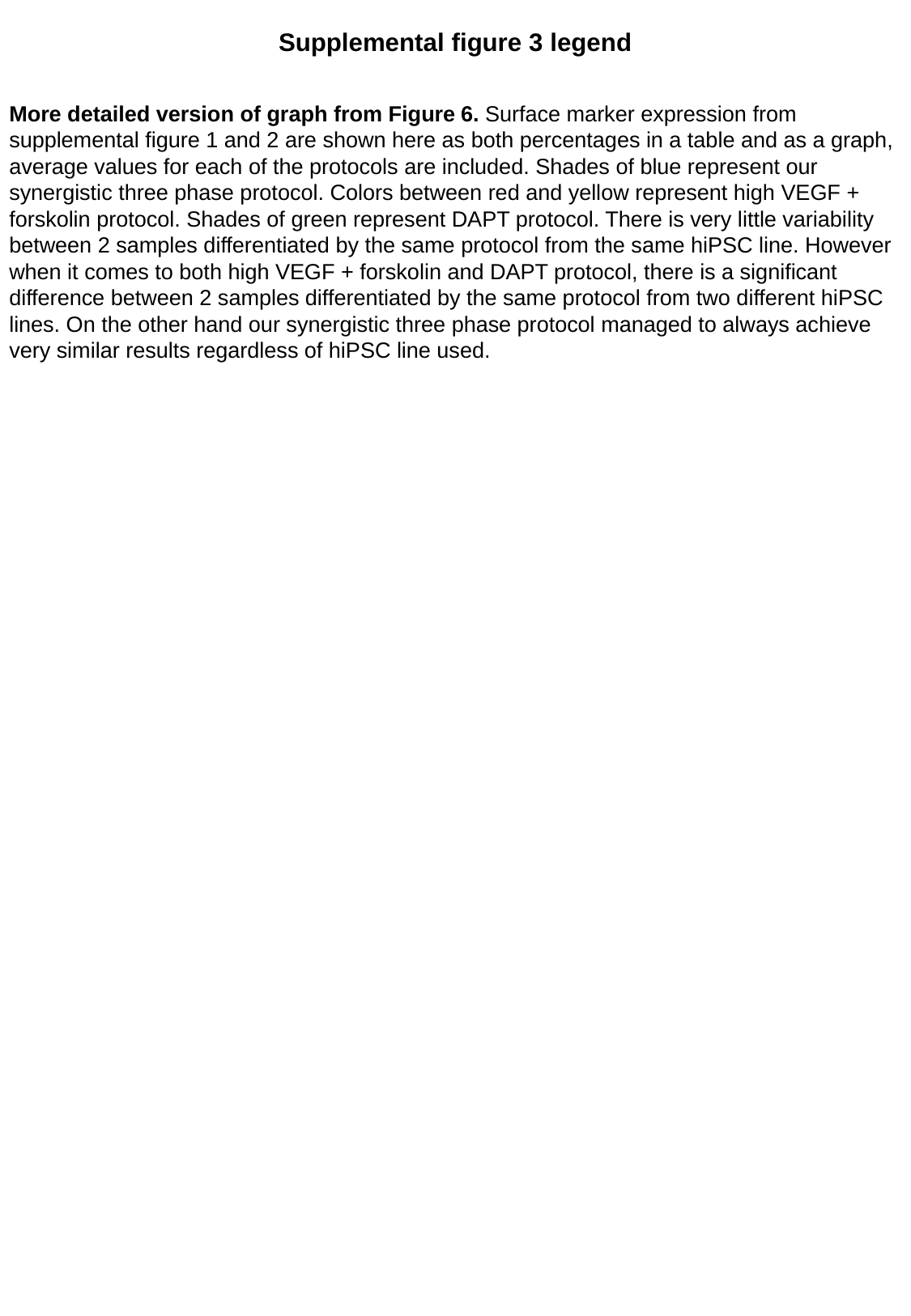

Supplemental figure 3 legend
More detailed version of graph from Figure 6. Surface marker expression from supplemental figure 1 and 2 are shown here as both percentages in a table and as a graph, average values for each of the protocols are included. Shades of blue represent our synergistic three phase protocol. Colors between red and yellow represent high VEGF + forskolin protocol. Shades of green represent DAPT protocol. There is very little variability between 2 samples differentiated by the same protocol from the same hiPSC line. However when it comes to both high VEGF + forskolin and DAPT protocol, there is a significant difference between 2 samples differentiated by the same protocol from two different hiPSC lines. On the other hand our synergistic three phase protocol managed to always achieve very similar results regardless of hiPSC line used.

## Slide 7
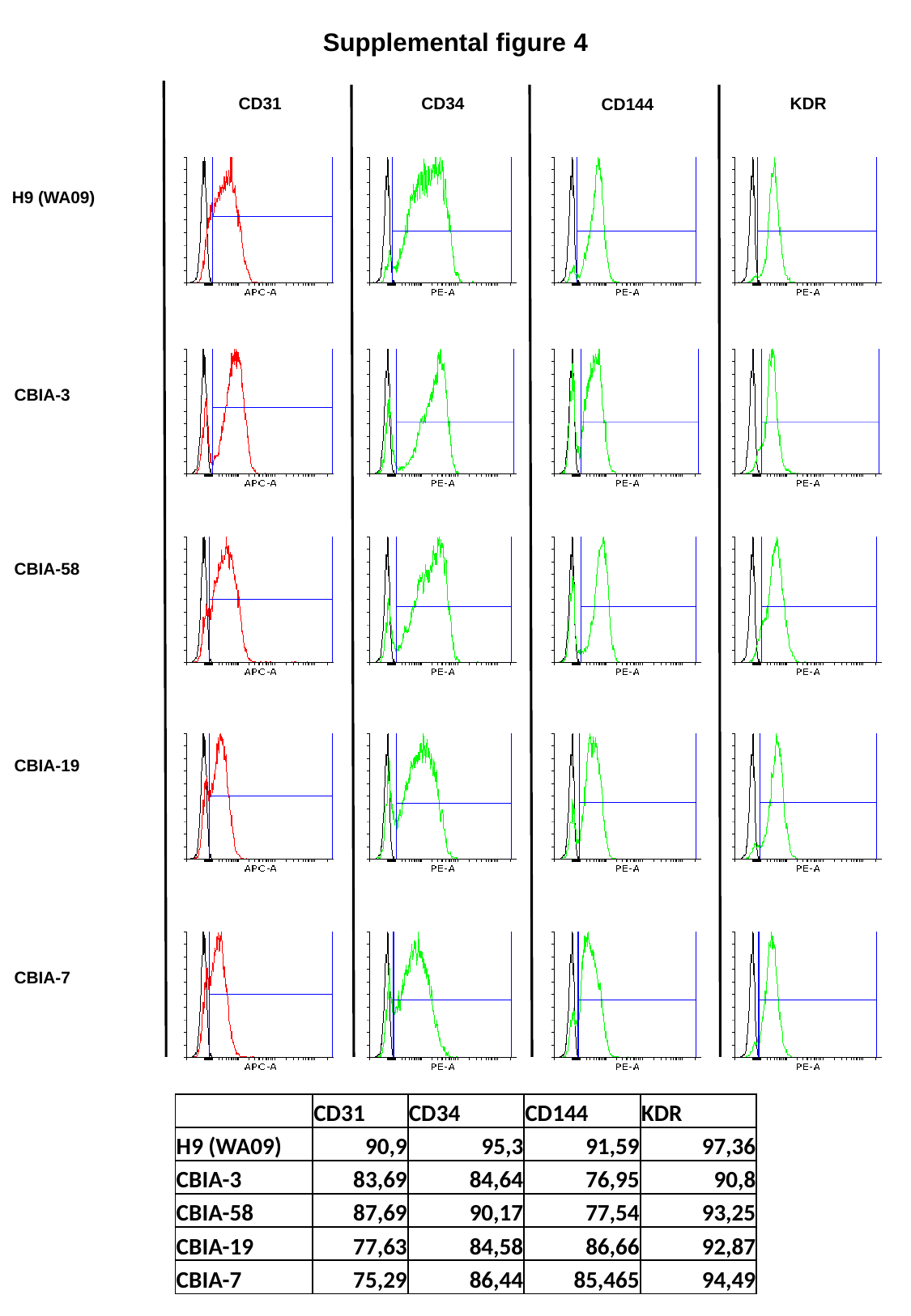

Supplemental figure 4
CD31
CD34
KDR
CD144
H9 (WA09)
CBIA-3
CBIA-58
CBIA-19
CBIA-7
| | CD31 | CD34 | CD144 | KDR |
| --- | --- | --- | --- | --- |
| H9 (WA09) | 90,9 | 95,3 | 91,59 | 97,36 |
| CBIA-3 | 83,69 | 84,64 | 76,95 | 90,8 |
| CBIA-58 | 87,69 | 90,17 | 77,54 | 93,25 |
| CBIA-19 | 77,63 | 84,58 | 86,66 | 92,87 |
| CBIA-7 | 75,29 | 86,44 | 85,465 | 94,49 |

## Slide 8
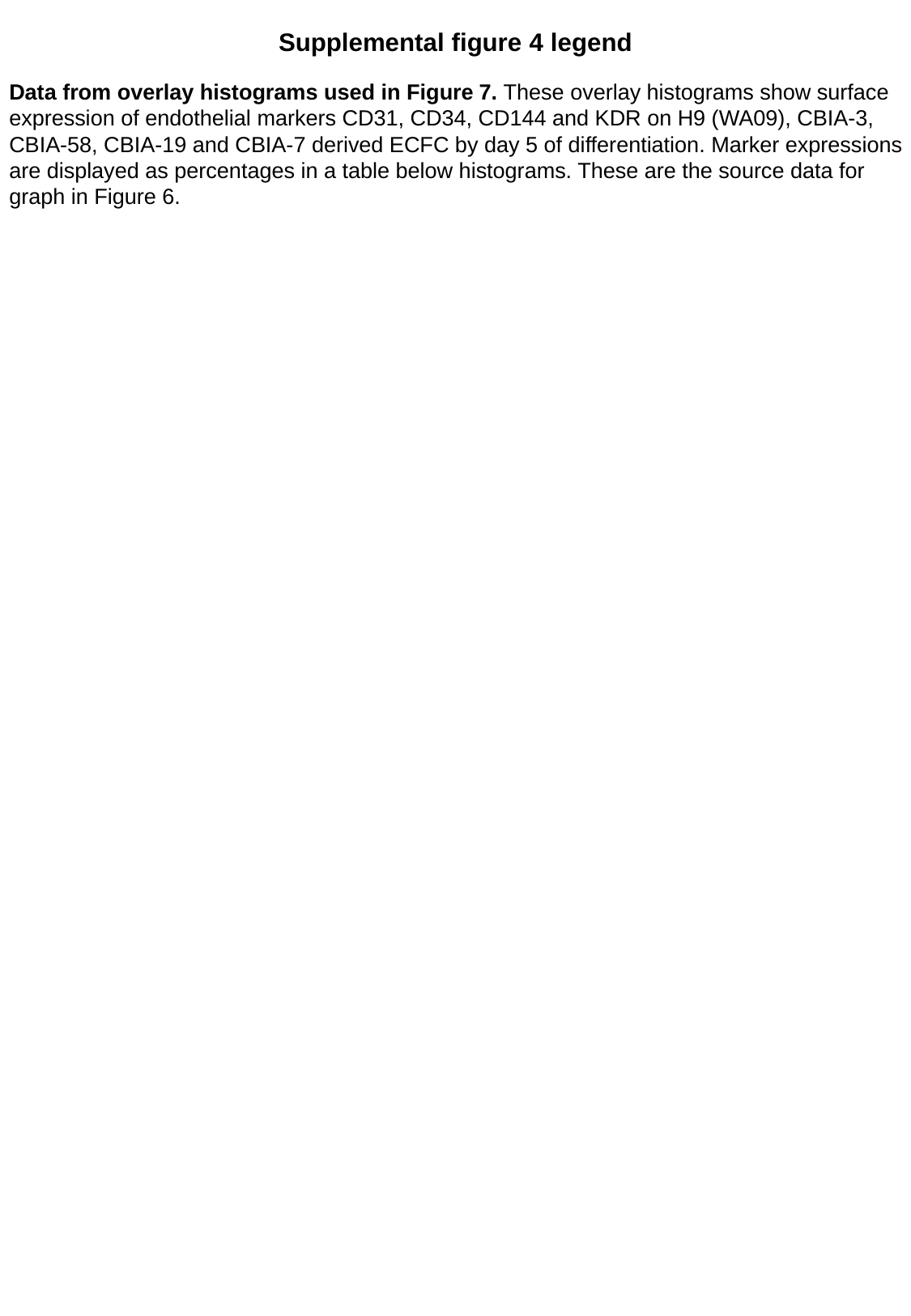

Supplemental figure 4 legend
Data from overlay histograms used in Figure 7. These overlay histograms show surface expression of endothelial markers CD31, CD34, CD144 and KDR on H9 (WA09), CBIA-3, CBIA-58, CBIA-19 and CBIA-7 derived ECFC by day 5 of differentiation. Marker expressions are displayed as percentages in a table below histograms. These are the source data for graph in Figure 6.

## Slide 9
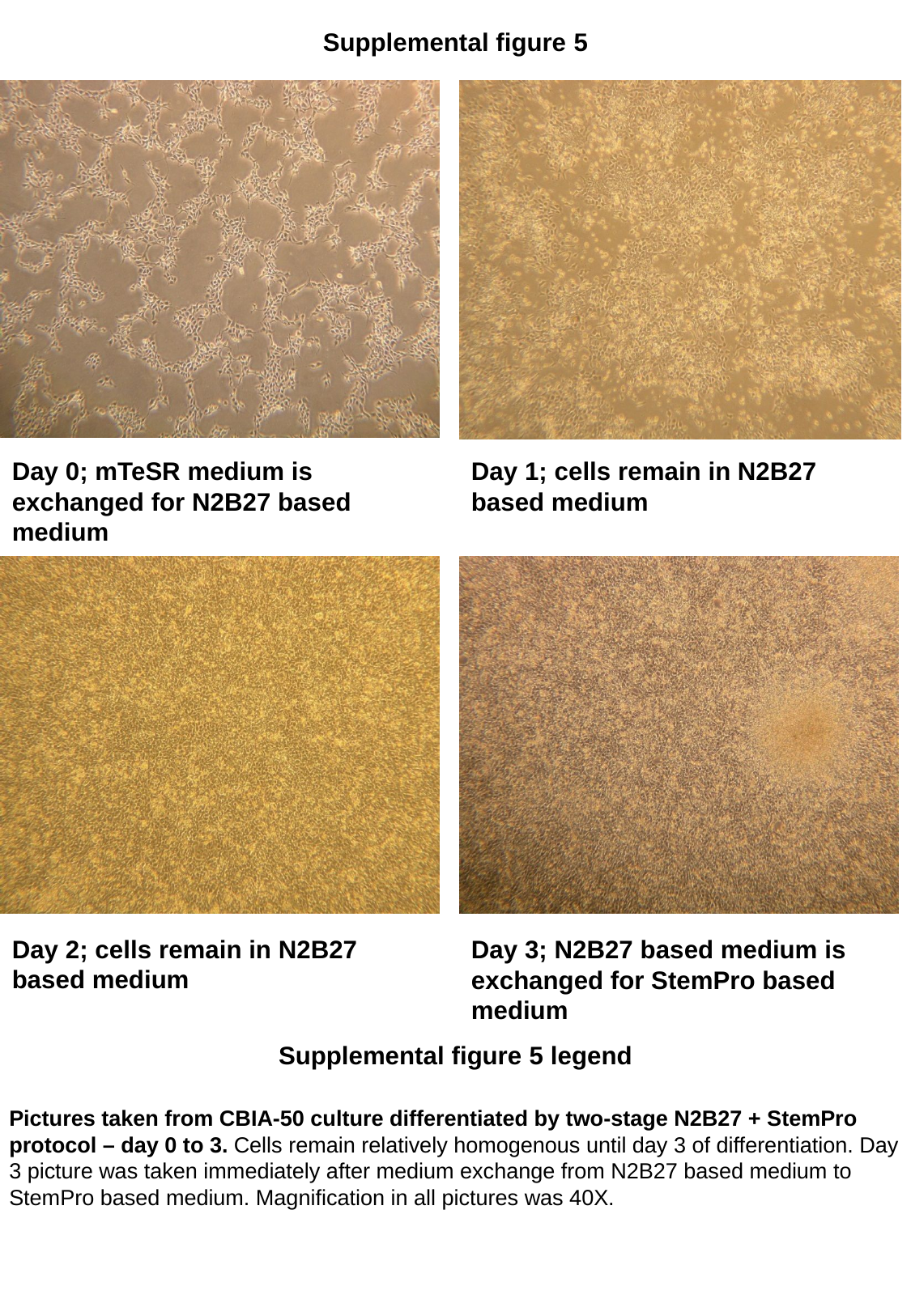

Supplemental figure 5
Day 0; mTeSR medium is exchanged for N2B27 based medium
Day 1; cells remain in N2B27 based medium
Day 2; cells remain in N2B27 based medium
Day 3; N2B27 based medium is exchanged for StemPro based medium
Supplemental figure 5 legend
Pictures taken from CBIA-50 culture differentiated by two-stage N2B27 + StemPro protocol – day 0 to 3. Cells remain relatively homogenous until day 3 of differentiation. Day 3 picture was taken immediately after medium exchange from N2B27 based medium to StemPro based medium. Magnification in all pictures was 40X.

## Slide 10
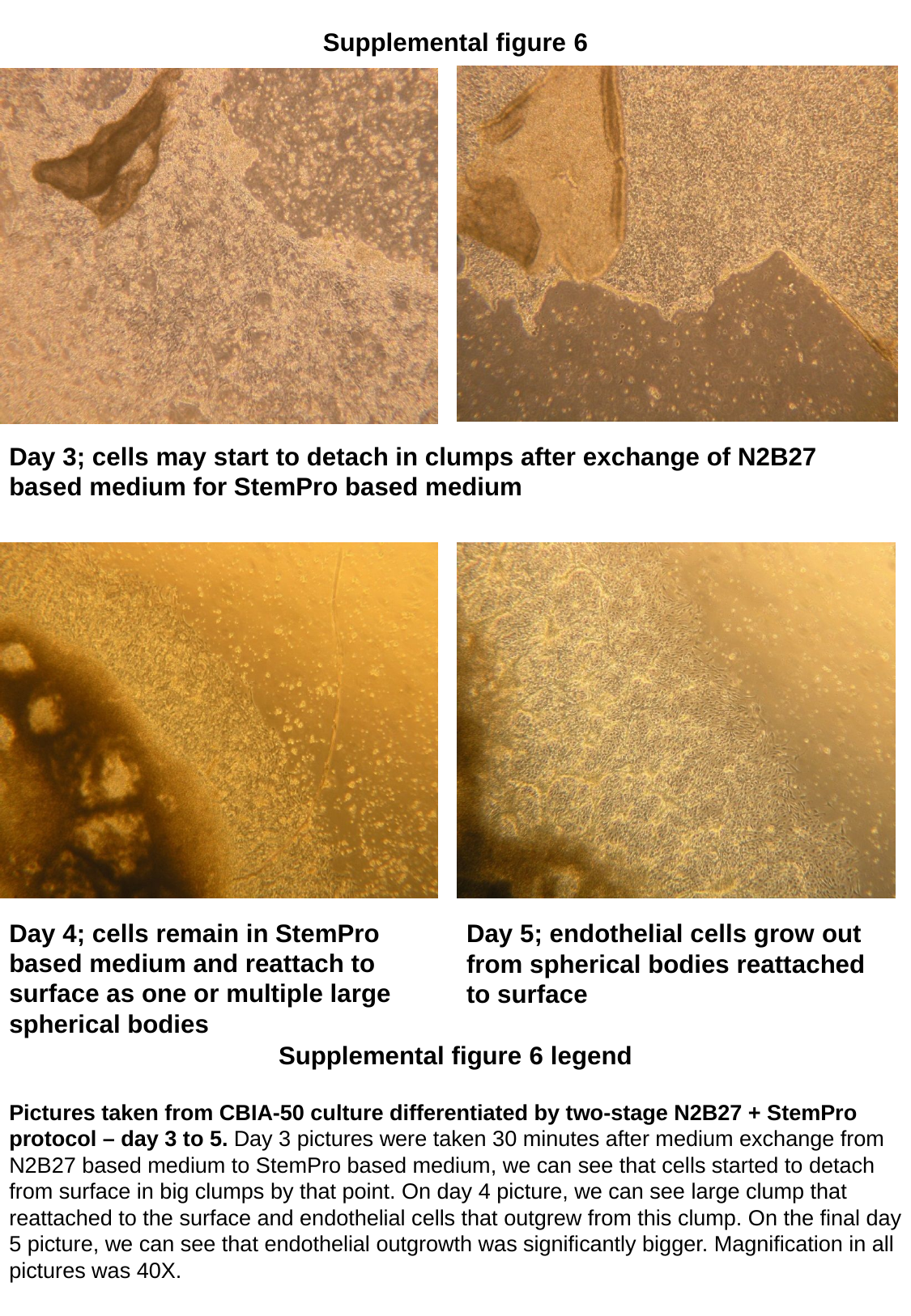

Supplemental figure 6
Day 3; cells may start to detach in clumps after exchange of N2B27 based medium for StemPro based medium
Day 4; cells remain in StemPro based medium and reattach to surface as one or multiple large spherical bodies
Day 5; endothelial cells grow out from spherical bodies reattached to surface
Supplemental figure 6 legend
Pictures taken from CBIA-50 culture differentiated by two-stage N2B27 + StemPro protocol – day 3 to 5. Day 3 pictures were taken 30 minutes after medium exchange from N2B27 based medium to StemPro based medium, we can see that cells started to detach from surface in big clumps by that point. On day 4 picture, we can see large clump that reattached to the surface and endothelial cells that outgrew from this clump. On the final day 5 picture, we can see that endothelial outgrowth was significantly bigger. Magnification in all pictures was 40X.
